# Dynamic magnetic resonance imaging of the female pelvic floor—a pictorial review

**DOI:** 10.1186/s13244-019-0687-9

**Published:** 2019-01-28

**Authors:** João Cunha Salvador, Mónica Portela Coutinho, José Marques Venâncio, Bárbara Viamonte

**Affiliations:** 1 Department of Radiology, Instituto Português de Oncologia de Lisboa Francisco Gentil, Rua Prof. Lima Basto, Lisboa, Portugal; 2 Department of Radiology, Hospital Universitário de São João, Centro Hospitalar São João, Porto, Portugal

**Keywords:** Female pelvic floor, Magnetic resonance defecography, Diagnostic imaging

## Abstract

Pelvic floor dysfunctions represent a range of functional disorders that frequently occur in adult women, carrying a significant burden on the quality of life, and its incidence tends to increase attending to the expected aging of the population. Pelvic floor dysfunctions can manifest as incontinence, constipation, and prolapsed pelvic organs. Since pelvic floor weakness is frequently generalized and clinically underdiagnosed, imaging evaluation is of major importance, especially prior to surgical correction. Given some interobserver variability of soft-tissue measurements, MR defecography allows a noninvasive, radiation-free, multiplanar dynamic evaluation of the three pelvic compartments simultaneously and with high spatial and temporal resolution. Both static/anatomic and dynamic/functional findings are important, since pelvic disorders can manifest as whole pelvic floor weakness/dysfunction or as an isolated or single compartment disorder. Imaging has a preponderant role in accessing pelvic floor disorders, and dynamic MR defecography presents as a reliable option, being able to evaluate the entire pelvic floor for optimal patient management before surgery. The purpose of this article is to address the female pelvic anatomy and explain the appropriate MR Defecography protocol, along with all the anatomic points, lines, angles, and measurements needed for a correct interpretation, to later focus on the different disorders of the female pelvic floor, illustrated with MR defecography images, highlighting the role of this technique in accessing these pathologic conditions.

## Key points

- The pelvic floor works as a single entity, and pelvic weakness is frequently generalized, affecting more than one compartment.
- Both the fluoroscopic cystocolpodefecography and MR defecography with rectal contrast opacification are appropriate and with equal rating scales for evaluation of pelvic floor disorders of the different compartments, with preference for MR defecography in assessing defecatory dysfunctions.
- The pelvic floor is divided into three anatomic compartments: anterior, middle, and posterior, connected by structures responsible for the pelvic support: craniocaudally, these are respectively the endopelvic fascia, the pelvic diaphragm, and the urogenital diaphragm.
- The first and most important line is the pubococcygeal line (PCL), a straight line connecting the inferior border of the pubic symphysis to the last coccygeal joint, representing the plane of attachment of the pelvic floor muscles, used as reference for measuring organ prolapses and drawn in the midline sagittal plane.
- Although prolapses may coexist with relaxation/laxity of the pelvic support structures, and themselves may induce functional disorders from external compression, pelvic prolapses can be independent from functional disorders.

## Introduction

Functional disorders of the pelvic floor refer to a group of medical conditions that affect the ligaments, fasciae, and muscles that together support the pelvic organs [1]. These debilitating conditions are relatively common, predominantly affecting older women, being estimated in more than 15% of multiparous women [2], and the demand for services related to treatment of this conditions is estimated to rise 45% from 2000 to 2030 based on the aging of the population [3]. Since these pelvic dysfunctions manifest as organ prolapse and/or urinary and defecatory dysfunctions, carry a significant burden in the quality of life.

Risk factors include female gender, pregnancy, multiparity, pelvic surgery, obesity, menopause, advanced age, connective tissue disorders, smoking, and any condition that may increase intraabdominal pressure, such as chronic pulmonary obstructive disease [4–7].

Traditionally, these pelvic disorders have been separated into three different groups, following both the anatomic division of the pelvic floor into anterior, middle, and posterior compartments, and the medical specialization into urology, gynecology, and proctology. Although this compartmentalization is relevant to systematize these conditions, it is also in part responsible for the frequent therapeutic failure associated with these surgical procedures [8], because the pelvic floor functions as a single entity, and pelvic weakness frequently is generalized, affecting more than one compartment [9–11], even though it may be clinically silent. Also, clinical evaluation still underestimates or misdiagnoses pelvic organ prolapses in as much as 45–90% of cases [10]. This becomes even more important when surgical intervention is considered, especially when a single compartment correction is done. Recurrence rates range from 10 to 30% in these patients [12], mostly related to other pelvic compartments that were not initially assessed or repaired. It is estimated that 30% of all the surgeries performed in these women are reoperations after unsuccessful treatments [2]. Nowadays, the surgical approach is to perform all the necessary corrections in one intervention [8] in order to prevent future relapse or exacerbation of other pelvic dysfunctions that may be initially associated. These facts impose the need for imaging evaluation of this complex and intricate anatomy for an accurate pretreatment diagnosis, specially before a surgical approach is considered.

Dynamic cystocolpodefecography remains a useful technic for functional assessment of the pelvic organs [8], being easily available, and respecting a physiological sitting position. However, following the first report by Yang et al. on dynamic MR protocol for evaluation of pelvic floor prolapse [13], subsequent studies have proven the reliability of this technic in accessing pelvic floor disorders. Although not as widely available as fluoroscopy, MR defecography presents several advantages in regard to its alternative: it is less invasive, less time consuming, radiation-free and displays a simultaneous multiplanar dynamic evaluation of the three pelvic compartments, with high spatial and temporal resolution. Despite some interobserver variability, mainly on measuring soft tissue parameters [14], MR defecography has been shown to be a superior method for evaluation of posterior compartment disorders [15, 16] and, regarding pelvic organ prolapses, has been proven to be reliable and with similar effectiveness as fluoroscopy [15–17]. Most of the time, it is executed in the supine position, lacking the physiological evacuation posture and bypassing the gravity effect, although new open-magnet units are increasingly available. Different studies demonstrate better and greater depiction of pelvic floor laxity on the sitting position, as well as rectal intussusception and usually better grading of different pelvic prolapses. However, for clinical and relevant findings during pelvic floor assessment, both technics show equivalent sensitivity [17–20].

Recently, the American College of Radiology (ACR) published the new Appropriateness Criteria for Pelvic Floor Dysfunction, stating that both the fluoroscopic cystocolpodefecography and MR defecography with rectal contrast opacification are appropriate and with equal rating scales for evaluation of pelvic floor disorders of the different compartments, with preference for MR defecography in assessing defecatory dysfunctions [21]. In anal incontinence, both endorectal ultrasound and pelvic MRI are preferred for assessing the anal sphincter, but it is indicated that MR defecography should be considered for evaluation of the entire pelvic floor in order to assess both the pelvic musculature and other possible associated abnormalities [21]. It is also favored, when possible, MR defecography in an open-magnet unit, to respect the normal physiologic position. Also recently, both the European Society of Urogenital Radiology (ESUR) and the European Society of Gastrointestinal and Abdominal Radiology (ESGAR) published a conjoined article regarding recommendations for pelvic floor dysfunction imaging, specifying the various indications where MR defecography is appropriate, with high levels of agreement (85–92%) between the authors regarding assessment of rectal outlet obstruction, rectocele, recurrent pelvic organ prolapse, and anismus, and at least moderate agreement (54–77%) in all other pelvic floor pathologies [22].

Although this article focuses on female pelvic floor anatomy and pathology, it is important to realize that, while much less frequent, pelvic floor disorders are also present in men which, attending to lesser risk factors (hormonal or traumatic), affect mainly the posterior compartment and are due mainly to functional dysfunctions.

## MR defecography protocol

Until recently, there has been no international recommendations concerning the adequate protocol of MR defecography, namely the need for anatomic/static sequences and dynamic ones, as well regarding about opacification of the rectum, vagina, and bladder or previous administration of oral contrast. That is the reason why so many studies show different MR sequences for the same type of study, and also different kinds of opacification for the rectum. After some studies about different rectal contrasts with different viscosities [23, 24] showing no significant differences in the evaluation of the posterior pelvic compartment, studies revealing the relevance of the evacuation phase [16], and also studies evaluating the feasibility of this exam in a 3-T MR closed-unit [25], the conjoined article of both ESUR and ESGAR from 2017 stated the necessary requisites and the basic MR defecography sequences needed for an adequate pelvic floor evaluation: the study should be made in at least a 1.5-T MR closed-unit with a phased array coil, with the patient in supine and with the knees elevated (proven to facilitate both straining and evacuation maneuvers); 120–250 cc of ultrasound gel is recommended for rectum opacification, no need for previous oral contrast administration neither opacification of the vagina, and the bladder should be half-full; the basic protocol should include anatomic high-resolution T2-weighted imaging (T2W) sequences in the axial, coronal, and sagittal planes of the pelvis, and dynamic sequences during straining, squeezing, and evacuation with steady state or balanced state free precession sequences [22]. It is vital to emphasize the importance of both the static and dynamic sequences in the evaluation of the patient, because with the conjoined assessment, it is possible to discriminate the different causes for the specific pelvic dysfunction, originating from muscular, fascial, or mixed origin [26]. It is also never enough to highlight the importance of the evacuation phase on the assessment of pelvic floor disorders, a sequence proven to be more sensitive in identifying and grading pelvic organ prolapses, pelvic floor descents, and rectal intussusceptions [16, 27].

Although these recommendations refer the preferred supine position with bended knees for the closed-magnet MR defecography study, lateral decubitus is also a position proven to be effective [28, 29]. In fact, a recent study demonstrated that lateral decubitus is more suited for evaluation of the evacuation phase, as the patients were more able to comply and cooperate in this maneuver, while also showing no changes in the evaluation of the descent of the pelvic floor [29].

At this hospital unit, MRI defecography protocol consists of three static/anatomic sequences and four dynamic/functional ones, both in a 1.5-T and 3-T MR closed-unit, following the ESUR recommendations stated above, adding only a TrueFISP dynamic sequences at rest, which we find helpful when comparing the mobility of the pelvic floor in the different dynamic states. Table 1 summarizes the protocol in detail.

**Table 1:** MR defecography protocol. FOV, field of view; TE, echo time; TR, repetition time

Pulse sequence	Imaging plane	TR/TE (msec)	FOV (cm)	Section thickness (mm)	Matrix	Study phase
T1-weighted localizer	–	–	Large	–	–	Rest
T2-weighted turbo spin-echo	Axial	6220/100	220	4	288/320	Rest
T2-weighted turbo spin-echo	Sagittal	4000/100	220	4	202/320	Rest
T2-weighted turbo spin-echo	Coronal	4860/100	240	4	288/320	Rest
TrueFISP	Midline Sagittal	4.3/1.85	300	10	320/320	Rest
TrueFISP	Midline Sagittal	4.3/1.85	300	10	320/320	Squeezing
TrueFISP	Midline Sagittal	4.3/1.85	300	10	320/320	Straining/Valsalva
TrueFISP	Midline Sagittal	4.3/1.85	300	10	320/320	Defecation

Patient cooperation is of maximum importance and usually they are very motivated. However, it is important to remember that, from the patient’s point of view, this is an awkward and embarrassing exam, even though it is done privately inside a closed-unit MR, because the supine position is not the typical posture of evacuation and it is not a clean study, because during the evacuation phase the ultrasound gel is expelled. The more information the patient gets about our aims and what we expect this study will clarify, the more cooperative the patient will become.

This protocol might be changed/adapted in the future, since new studies with other modalities that might add supplement information are undergoing, namely the diffusion tensor imaging with fiber tractography [30, 31] for evaluation of the pelvic musculature.

## Pelvic anatomy

**Fig. 1:**
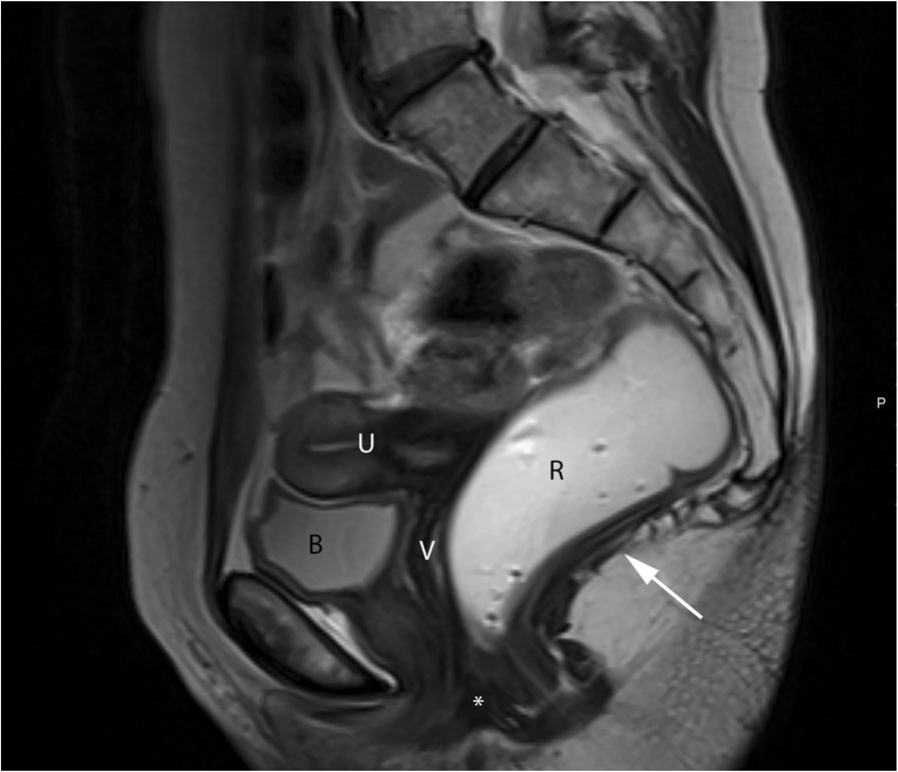
Normal female pelvic anatomy. Sagittal midline T2-TSE image demonstrating the perineal body (*) and the levator plate (white arrow). *B*, bladder; *U*, uterus; *V*, vagina; *R*, rectum

In order to correctly evaluate MRI defecography, knowledge of the pelvic anatomy is essential. Since these disorders are much more frequent on women, relevant female pelvic anatomy will be reviewed (Fig. 1).

**Fig. 2:**
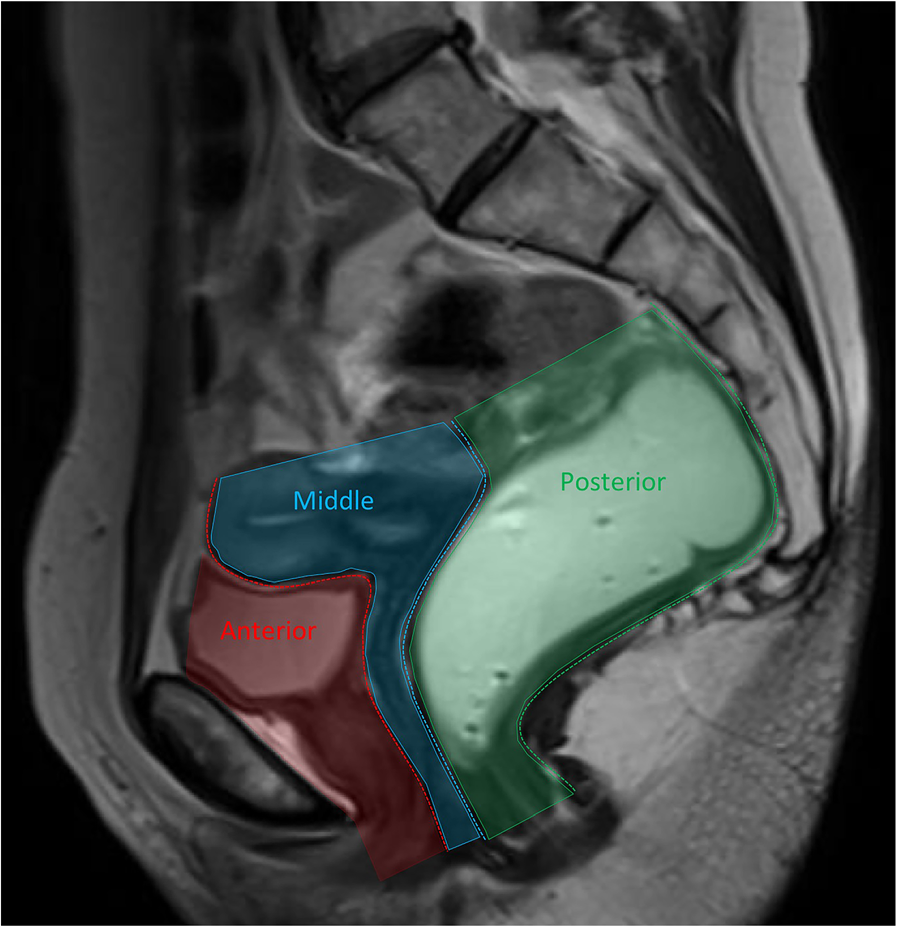
Pelvic compartments. Sagittal midline T2-TSE image representing the three pelvic compartments in different colors

As mentioned above, the pelvic floor is divided into three anatomic compartments: anterior, middle, and posterior. In the anterior compartment lies the bladder and the urethra; in the middle compartment the uterus, cervix, and vagina; and in the posterior compartment the rectum, anus, and anal sphincter (Fig. 2). Connecting these compartments there are three structures responsible for the pelvic support: craniocaudally, these are respectively the endopelvic fascia, the pelvic diaphragm, and the urogenital diaphragm. The last one is restricted to the anterior and middle compartments.

**Fig. 3:**
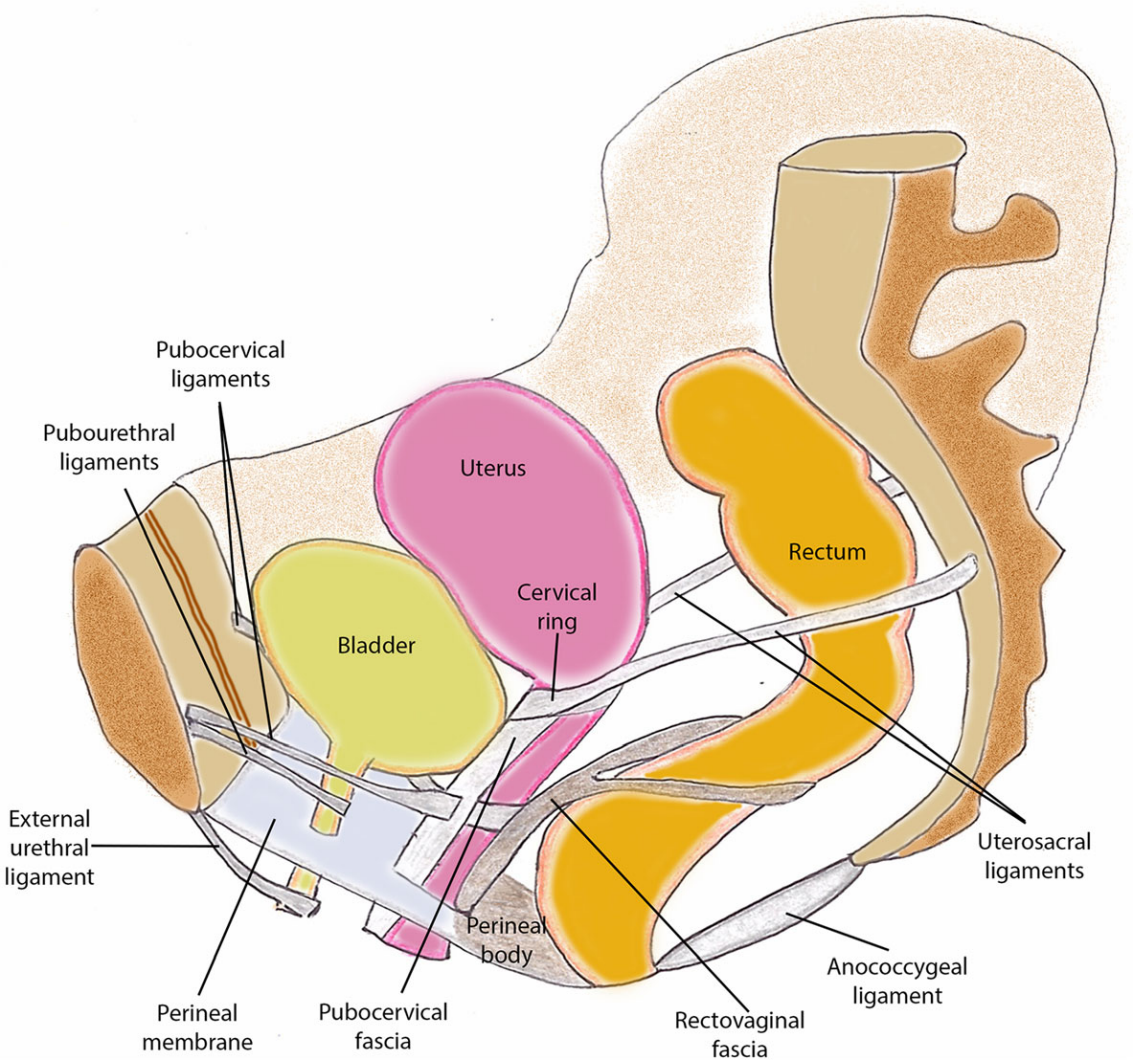
Representative image of the pelvic floor ligaments and their relative anatomical positions in a female model

The endopelvic fascia is a layer of connective tissue that surrounds and gives support to all pelvic organs, covering superiorly the levator ani muscle. In the anterior compartment, it gives rise to the pubocervical ligaments; the three groups of ligaments that support the urethra: periurethral, paraurethral, and pubourethral ligaments; and the pubovesical ligaments. In the middle compartment, it relates to the parametrium and paracolpium, where the cardinal ligaments of the cervix and the uterosacral ligaments are located. In the posterior compartment, its midline condensation originates the perineal body, in the anovaginal septum, serving as an anchor of support for multiple muscles and ligaments and preventing the expansion of the urogenital hiatus. Also, it continues as the rectovaginal fascia, between the posterior wall of the vagina and the anterior wall of the rectum. Lateral condensations of this endopelvic fascia that attach in the pelvic bones from the pubic symphysis to the spine of the ischium represent the tendinous arch, providing passive pelvic support and serving as a point of attachment of the levator ani muscle (Fig. 3). On MR images, only the urethral ligaments, the perineal body and sometimes the uterosacral ligaments can be identified. Others can be inferred only from anatomic defects that usually result in pelvic prolapses [1].

**Fig. 4:**
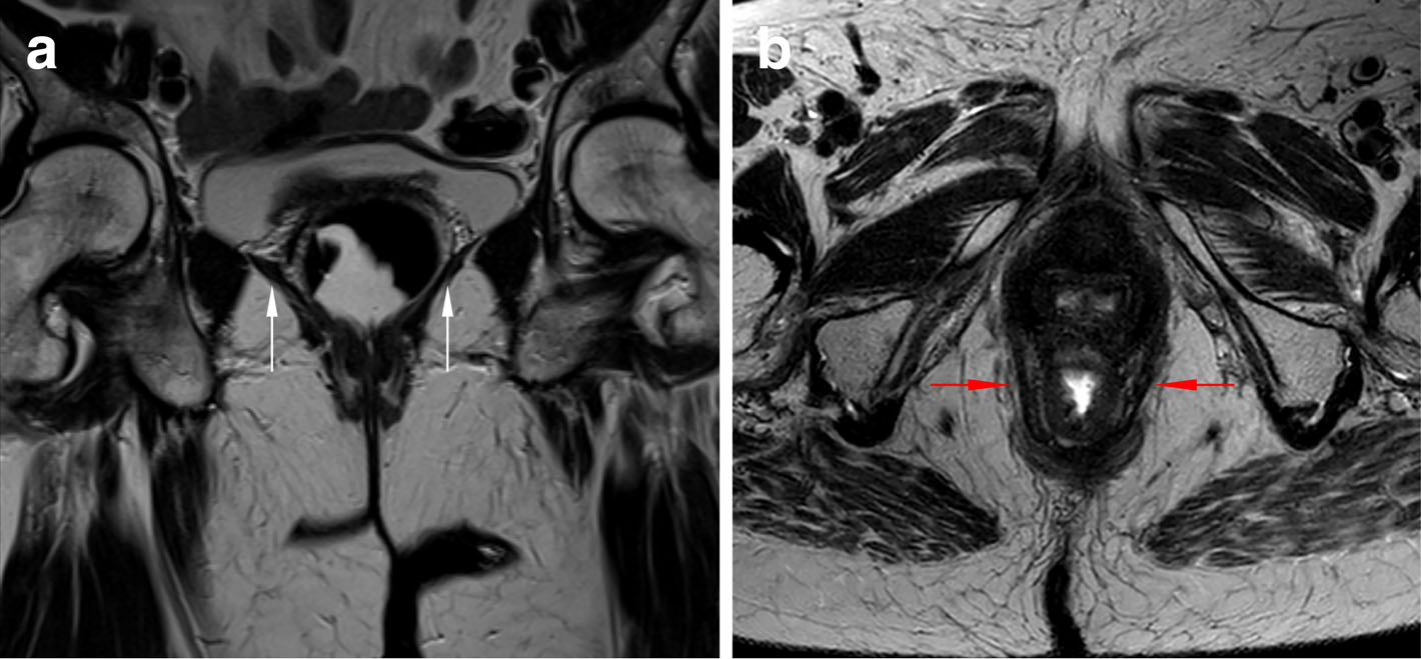
Pelvic muscles. (**a, b**) Coronal (**a**) and axial (**b**) T2-TSE images of the pelvic diaphragm, showing the iliococcygeus (white arrows) and puborectalis (red arrows) muscles

The pelvic diaphragm is located deep to the endopelvic fascia and consists of four muscles: ischiococcygeus, iliococcygeus, pubococcygeus, and puborectalis (the last three forming the levator ani muscle). These muscles, at rest, are tonically and simultaneously contracted, in order to provide pelvic floor tone, support the pelvic organs, and maintain continence [32]. The two most important and easily identified on MR images are the iliococcygeus and puborectalis muscles. The first one fans out laterally from the external anal sphincter and inserts in the posterior part of the tendinous arch (seen on coronal planes) (Fig. 4a). Posteriorly, in the midline, a condensation of this muscle originates the levator plate. The puborectalis muscle has a U-shape sling-like appearance, inserting in the parasymphiseal area and going around the posterior wall of the rectum (seen on axial planes), defining the anorectal junction (Fig. 4b).

The urogenital diaphragm is located anteriorly to the anorectum and inferiorly to the pelvic diaphragm. It is a triangular structure between the ischial bones, formed by the deep perineal transverse muscle and the superior and inferior fascia of the urogenital diaphragm, enclosing the sphincter urethrae and the deep perineal space.

### MR defecography interpretation and normal findings

There is no universally accepted method for MR defecography interpretation. Several points and lines for measuring and staging pelvic organ prolapse at MR imaging have been proposed; however, most agree that PCL line has the highest inter- and intra-observer reliability of MRI measurements compared to all proposed reference lines [21, 22].

**Fig. 5:**
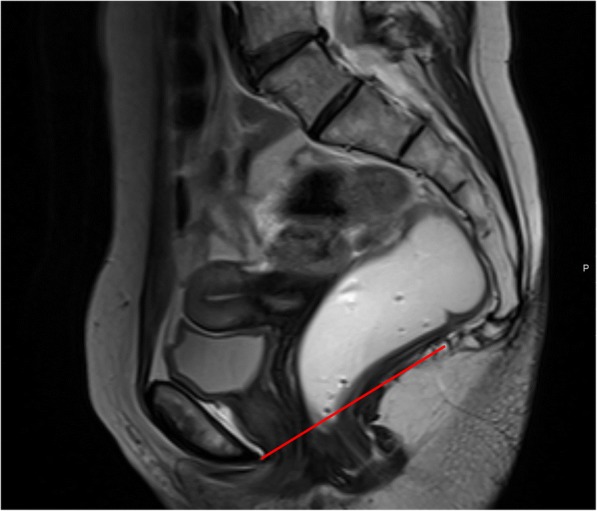
Pubococcygeal line (PCL). Sagittal midline T2-TSE image with the PCL represented (red line) connecting the inferior border of the pubic symphysis and the last coccygeal joint

The first important line is the pubococcygeal line (PCL), a straight line connecting the inferior border of the pubic symphysis to the last coccygeal joint (Fig. 5). This represents the plane of attachment of the pelvic floor muscles, used as reference for measuring organ prolapses and drawn in the midline sagittal plane [33–36]. In healthy asymptomatic patients, the movement of pelvic organs in any phase of the MR defecography is minimum and never more than 1 cm below this line [13] always measured perpendicular in relationship to the PCL.

**Fig. 6:**
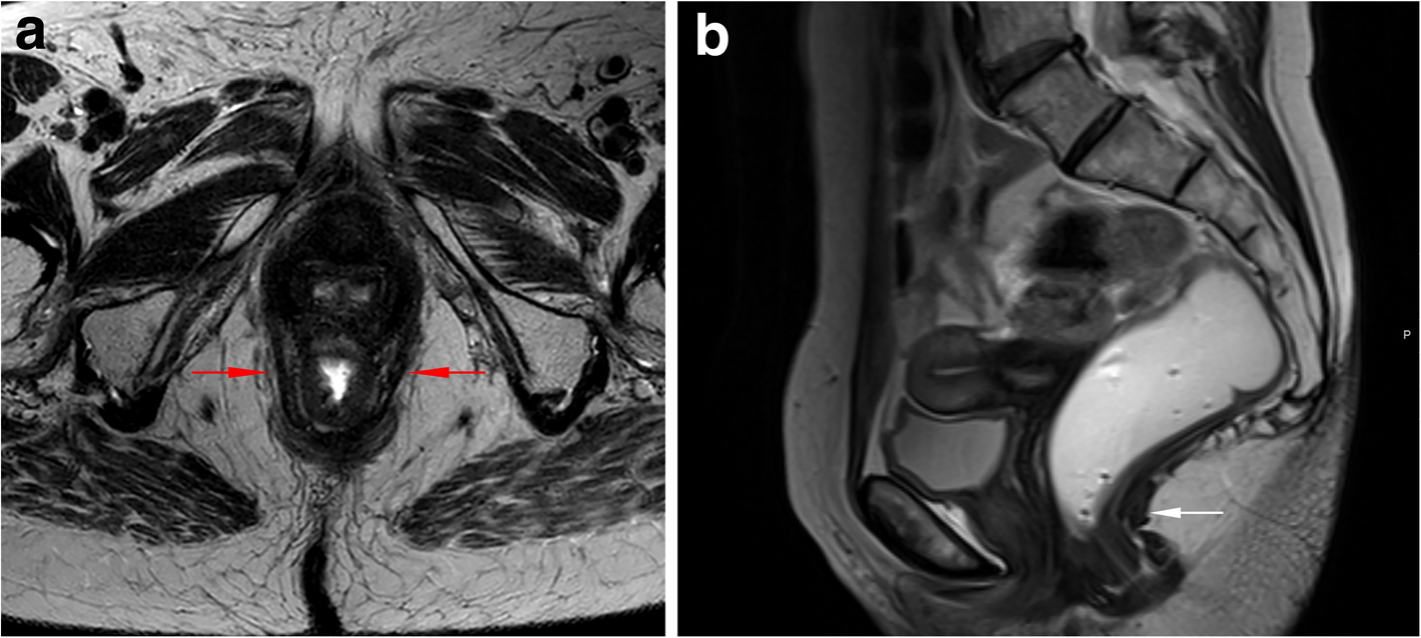
Anorectal junction (ARJ). (**a, b**) Axial T2-TSE image (**a**) at the plane of the puborectalis muscle (red arrows), the same plane of the ARJ. Sagittal T2-TSE image **(b)** showing the posterior wall of the ARJ (white arrow)

**Fig. 7:**
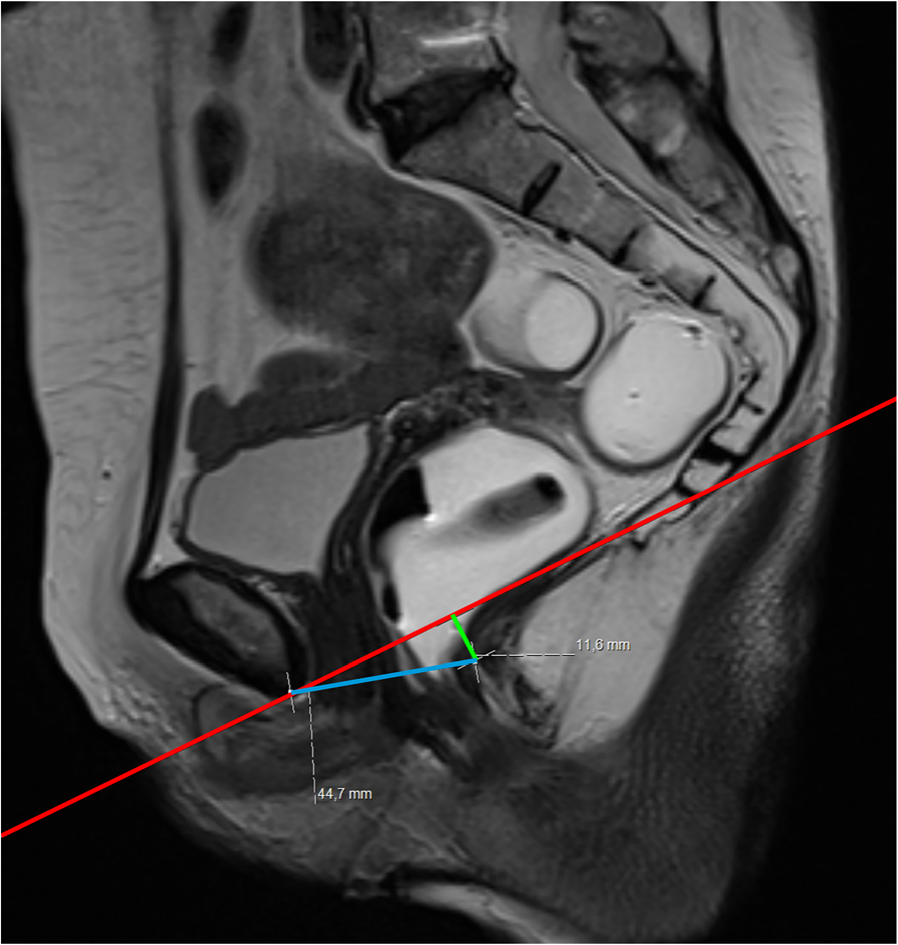
Normal H and M lines. Sagittal midline T2-TSE image demonstrating the normal measurements of the H (4.47 cm) and M (1.16 cm) lines. *Red line*: PCL; *blue line*: H line; *green line*: M line

The next important mark is the posterior wall of the rectum at the anorectal junction. This is the point where the puborectalis muscle slings around the rectum. It is easily individualized in the axial plane, when searching for the puborectalis muscle (Fig. 6a). In the midline sagittal plane, it is defined as the point of taper and angulation of the distal part of the rectum as it meets the anal canal [35], sometimes more difficult to access (Fig. 6b). To this anatomical point converge two important lines: H and M lines (Fig. 7). The H line, representing the anteroposterior width of the levator hiatus, connects this point with the inferior border of the pubic symphysis [33, 35]. The M line is drawn perpendicular to the PCL, intersecting the same point at the posterior wall of the anorectal junction, and stands for the vertical descent of the levator hiatus [33, 35]. Both these lines are dynamic, shrinking or elongating depending on the degree of contraction or relaxation of the pelvic floor muscles, respectively. Normal distances are considered not to exceed 5 cm and 2 cm, for the H and M line respectively [33, 35]. These two lines are important to grade the severity of pelvic floor relaxation.

**Fig. 8:**
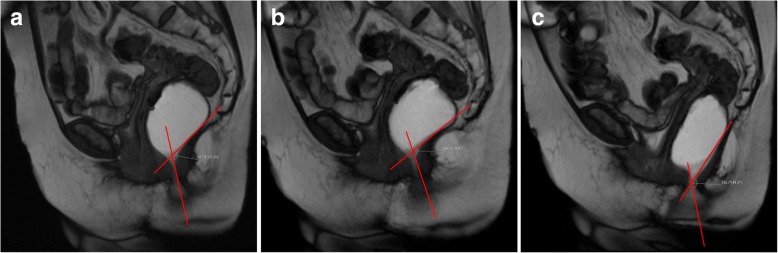
Normal anorectal angles. (**a**, **b**, **c**) Sagittal midline TrueFISP images at rest (**a**), during squeezing (**b**), and evacuation (**c**), with normal measurements of the ARA: 122° (**a**); 109° (**b**); 137° (**c**)

The posterior wall of the anorectal junction serves also as the apex of the anorectal angle, between the posterior border of the distal part of the rectum and the central axis of the anal canal. Normal measures obtained at rest are considered between 108° and 127° [18, 19, 34, 35] (Fig. 8). As the levator ani muscle contracts, the anorectum junction, vagina, and urethra are compressed anteriorly facing the pubic symphysis, decreasing the anorectal angle about 15° to 20°. When relaxing, the angle normally increases the same amount [18, 19]. The anorectal angle is important to access the pelvic diaphragm basal tonus and the ability to normally contract and relax.

**Fig. 9:**
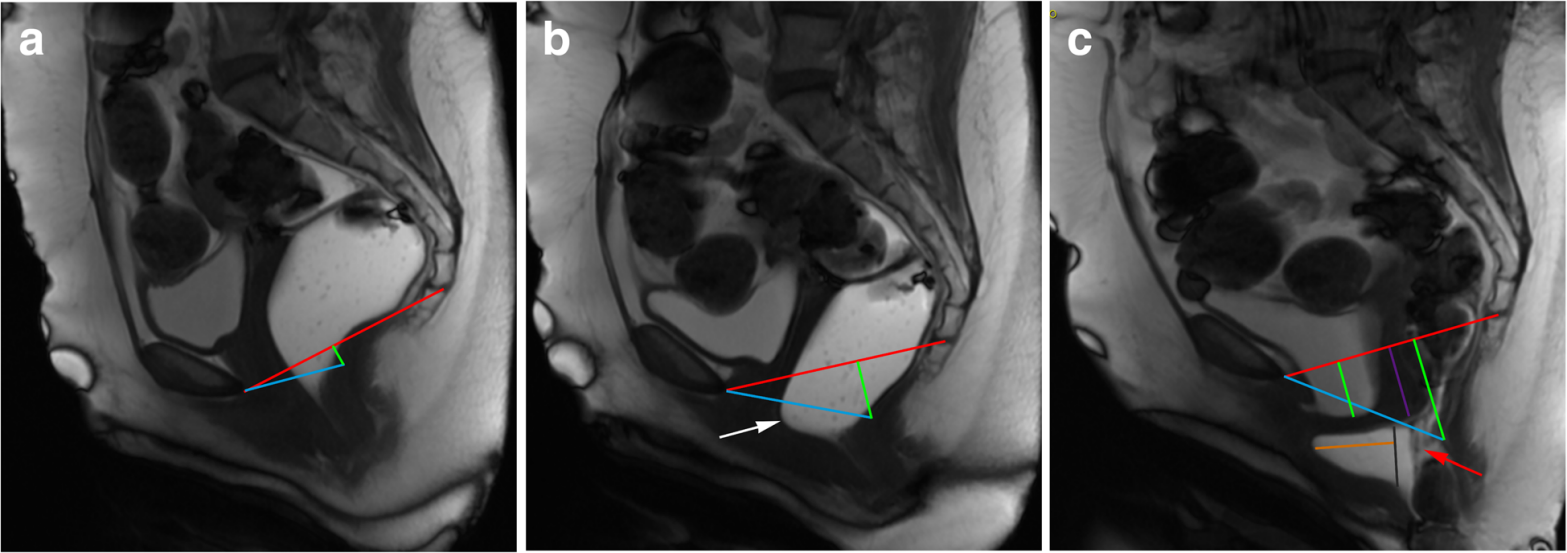
Importance of the evacuation phase. (**a**, **b**, **c**) Sagittal midline TrueFISP images at rest (**a**), during straining (**b**), and evacuation (**c**) of a patient with descending perineal syndrome with tricompartimental prolapse, anterior rectocele, and rectal mucosa intussusception. Note that, although in the straining phase (**b**) the H and M lines (blue and right green lines) are already above the normal values, indicating a descending perineal syndrome, and a mild bulge of the anterior rectal wall is seen (white arrow), only in the evacuation phase (**c**) the true pelvic floor disorder is adequately characterized, with a tricompartimental prolapse (cystocele, uterine prolapse, and rectal prolapse) the true size of the rectocele is documented (orange line: 4.8 cm) and the rectal mucosa intussusception is depicted (red arrow). *Left green line*: extent of cystocele; *purple line*: extent of uterine prolapse; *black line*: normal expected contour of the anterior anorectal wall; *red line*: PCL line

At last, a final statement related to grading pelvic organ prolapses and pelvic floor relaxation is needed. According to the ESUR/ESGAR recommendations, grading is based on the “rule of three”: mild grade relates with a pelvic organ prolapse or pelvic floor descent 3 cm or less below the PCL line; moderate grade between 3 cm and 6 cm below the PCL line; and severe grade when 6 cm or more below the PCL line [22]. However, this grading system is based solely on findings of the straining sequence. As cited above, the evacuation phase has been proven to be more sensitive in the identification of pelvic organ prolapses and also is associated with upgrading the severity of those prolapses [16, 27] (Fig. 9). Recently, Shawkat et al. showed again that looking at the pelvic floor descent on the straining phase may underestimate its degree [37]. Also, if this grading scale is applied during the evacuation phase, more asymptomatic patients may be diagnosed with descending perineal syndrome, and a new cutoff value of 4.5 cm below the PCL line for the posterior compartment evaluation is proposed [37], which may need additional investigation.

Normal findings in an MR defecography relate with the values of all the measurements cited above, namely the H and M lines, and the anorectal angle. Also, no pelvic organ prolapse is considered clinically significant if it does not descend more than 1 cm below the PCL line, and regarding the posterior compartment, a mild descent is considered within the normal range (less than 3 cm below the PCL line). A quick note related with anterior rectoceles is that its grading system is based on the “rule of two”, and a rectocele with less than 2 cm, more comprehensively described below, has no clinical significance and may be considered normal.

## Functional evaluation of the three compartments

Functional disorders of the pelvic floor may result from various causes. It is important to realize that, although prolapses may coexist with relaxation/laxity of the support structures, and themselves may induce functional disorders from external compression, pelvic prolapses can be independent from functional disorders [32]. Pelvic organ prolapses are defined as abnormal descent/bulge of the walls of the vagina, caused by protrusion of adjacent pelvic viscera [21]. This is a consequence of an anatomic defect in the support structures. Pelvic floor relaxation/laxity is triggered by lack of response and/or weakness of both the pelvic diaphragm and endopelvic fascia [32]. This presents clinically as dysfunction of the normal continence mechanisms and/or constipation, related to widening and descent of the pelvic floor.

### Anterior compartment disorders

Anterior compartment disorders, especially in the female patient, are always a clinical challenge, due to the nonspecific signs they usually present, such as urinary frequency, urinary urgency, incontinence, dysuria, or pelvic pain [38]. MR Defecography is, nowadays, the imaging modality of choice for the preoperative assessment of the anterior compartment dysfunctions [39]. The main role of this study is to detect the actual position and mobility of the bladder, the bladder neck, and the urethra [21], while it can also search for associated or other diseases of the lower urinary tract, such as urethral diverticulum, which in 60% of cases presents as urinary incontinence, mimicking other dysfunctions and usually being referenced for surgical treatment [40].

The main functional differential diagnosis in these patients is the presence of a cystocele, with or without associated urethral hypermobility, which presents as a spectrum of abnormalities with its associated range of symptoms.

**Fig. 10:**
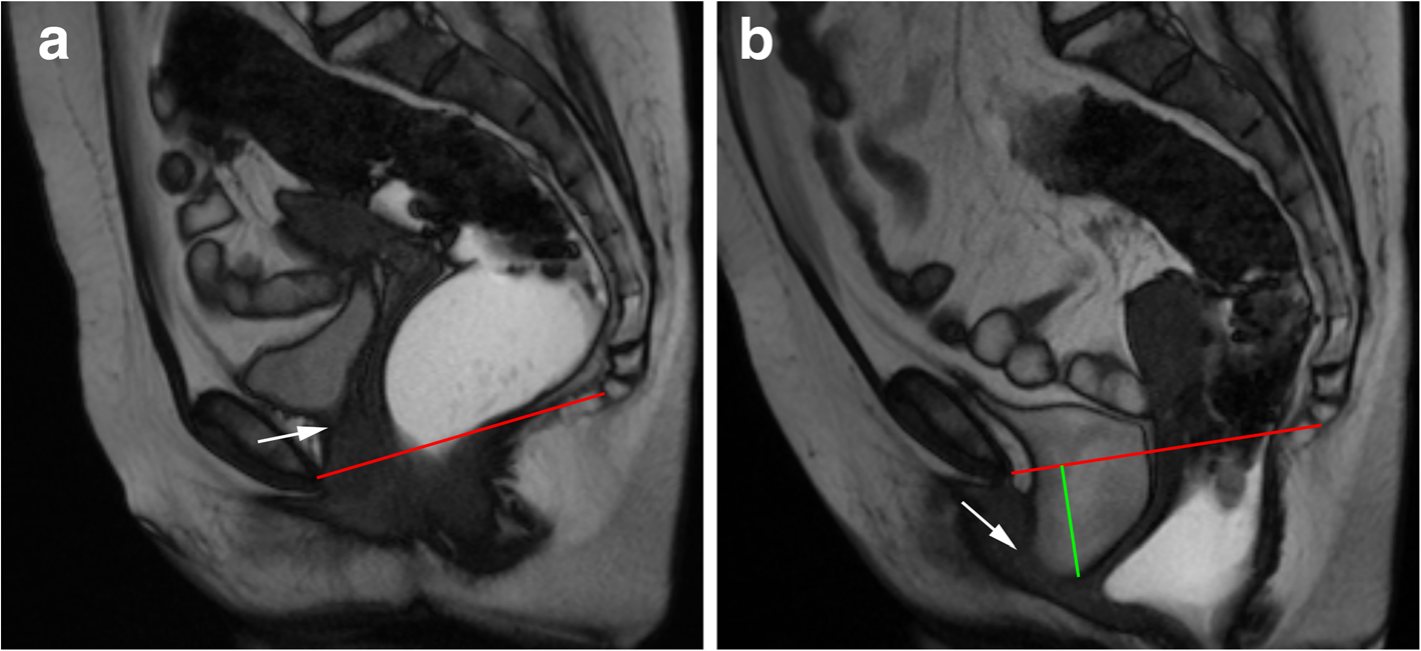
Moderate cystocele with urethral hypermobility. (**a**, **b**) Sagittal midline TrueFISP images at rest (**a**) and during evacuation (**b**), demonstrating descent of the bladder during evacuation (green line—3.8 cm) below the PCL (red line), as well as horizontalization of the urethral axis during defecation (white arrows). Note also an anterior rectocele in (**b**)

A cystocele is, by definition, an abnormal descent of the bladder, pushing posteriorly the anterior wall of the vagina [15]. Cystoceles may present as stress urinary incontinence and/or urinary retention, in the last case usually when there is a disproportional descent of the posterior wall of the bladder, resulting in kinking of the bladder neck [32, 41]. It is caused by a loss of support from the pubocervical fascia (part of the endopelvic fascia), due to tearing and/or stretching, [35] and is diagnosed by MR defecography when the most inferior aspect of the bladder is more than 1 cm below the PCL [15, 22, 35] (Fig. 10). Its severity relates with the distance between these two points of reference and is graded, just like other pelvic prolapses, as small, moderate, and severe, when less than 3 cm, between 3 and 6 cm and more than 6 cm below the PCL respectively [22, 42]. When the bladder prolapses, it occupies part of the width of the levator hiatus, pushing posterior and inferiorly both the vagina and the anorectal junction. Consequently, both H and M lines increase, related to stretching the levator ani muscle. Over time, this stretching may induce weakness and dysfunction of all the pelvic floor [33]. Cystoceles, like all pelvic prolapses, are better depicted in the late evacuation phase, due both to the increased abdominal pressure and the relaxation of the pelvic diaphragm [1] (Fig. 10b). When the posterior wall is disproportionally prolapsed, ureterohidronephrosis may coexist due to entrapment of the distal ureters by the pelvic musculature [32].

As mentioned above, cystoceles may coexist with urethral hypermobility. The normal urethra has a somewhat vertical orientation. Urethral hypermobility is defined as a clockwise rotation of the axis of the urethra, gaining a more horizontal orientation, especially when there is a rise in the intraabdominal pressure [35, 39]. It usually presents as stress urinary incontinence and is due to lack of support of the urethra, mainly from pubocervical ligaments and connective tissue around the vagina [15]. It is extremely important to assess separately the cystocele and the urethral hypermobility, because stress urinary incontinence may be masked by the coexistence of both and only noticed after a surgical cystocele correction [15, 32]. On MR defecography, straining and evacuation phases are the best sequences for the evaluation of urethral hypermobility, due to increased abdominal pressure: the proximal urethra moves inferiorly and the axis becomes more horizontal (Fig. 10). However, these findings should be integrated with the clinical evaluation, and only called relevant when accompanied by urinary incontinence, since it is a relative common finding in asymptomatic patients.

### Middle compartment disorders

In the pelvic middle compartment, disorders are related to uterine or cervical prolapse. Uterine prolapses can stay clinically unnoticed because the walls of the vagina function as a sac that contains the prolapsed organ [32], but can also manifest as dysfunction of the entire pelvic floor because, like stated above regarding cystoceles, the prolapsed uterus will stretch the pelvic hiatus, inducing weakness and dysfunction over time of both the endopelvic fascia and pelvic diaphragm [33]. In severe cases, where the prolapsed uterus extends beyond the vaginal introitus, known as procidentia, clinical evaluation of any coexistent pelvic prolapse gets difficult.

**Fig. 11:**
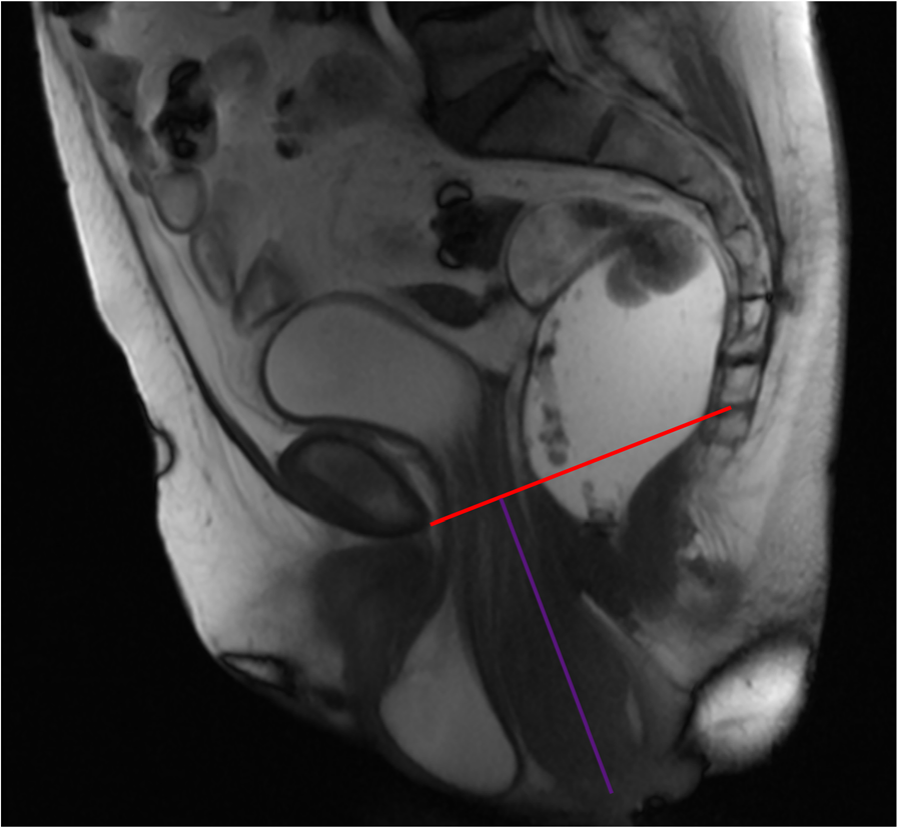
Uterine prolapse. Sagittal midline TrueFISP image at rest revealing an everted uterus filling of the vaginal space, which extends below the distal third of the vagina. *Red line*: PCL; *purple line*: extent of the uterine prolapse

A uterine prolapse is defined by a descent of the uterus and cervix into the vagina [8]. It is caused by defects of the multiple support structures that surround them, namely the pubocervical ligaments, parametrium and paracolpium, uterosacral ligaments, rectovaginal fascia, and perineal body, with special significance over the uterosacral ligaments that, when weakened, enable the cervix to move anteriorly, which subsequently allow retroversion and concomitant prolapse [32]. On MR defecography, it is diagnosed when the cervix is located 1 cm below the PCL [20, 35] (Fig. 11), using the distance between the PCL and the most anterior and inferior aspect of the cervix as reference for grading [1]: small when the distance is less than 3 cm, moderate when 3 to 6 cm, and severe when over 6 cm. The H and M lines are also usually increased due to widening of the levator hiatus caused by the interposed uterus, and the vagina acquires a more horizontal orientation [35].

In cases of previous hysterectomy, the vaginal apex should be located at least 1 cm above the PCL, using the most posterior and superior aspect of the vaginal vault [1]. Vaginal vault prolapse is frequently associated with other pelvic prolapses, most commonly an enterocele [8].

### Posterior compartment disorders

The posterior pelvic compartment houses a greater diversity of dysfunctions. It may clinically present as fecal incontinence or constipation, the last ones usually complaining of incomplete evacuation, excessive straining, and need for manual assistance during defecation [34].

**Fig. 12:**
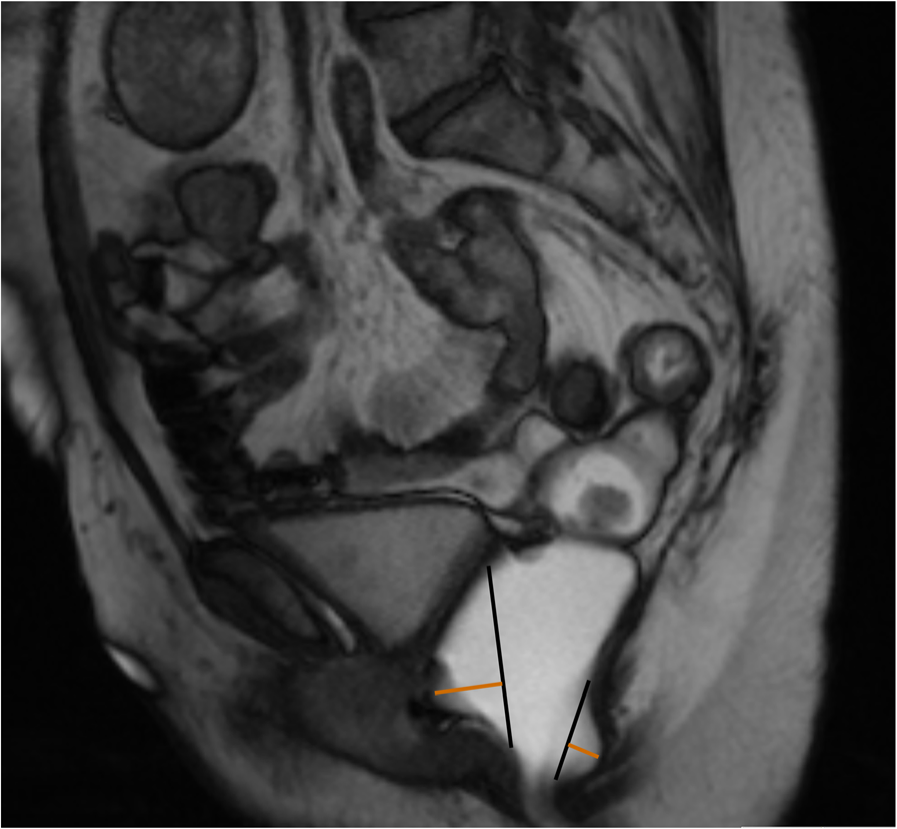
Small rectoceles. Sagittal midline TrueFISP image during evacuation, showing both anterior and posterior small rectoceles. *Black lines*: normal expected contours of the anorectal wall; *orange lines*: extent of the rectoceles: 13 mm anterior and 9 mm posterior

**Fig. 13:**
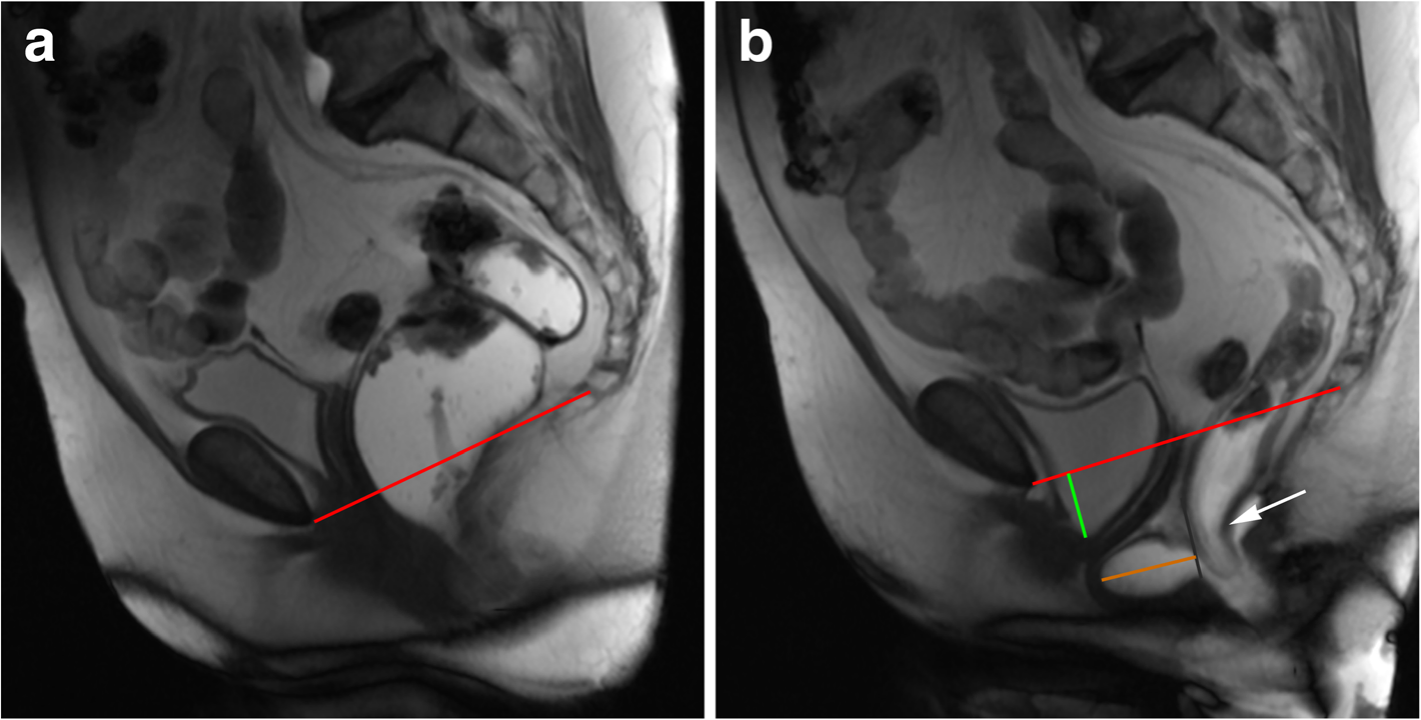
Moderate anterior rectocele. (**a**, **b**) Sagittal midline TrueFISP images at rest (**a**) and during evacuation (**b**) revealing a moderate anterior rectocele (orange line: 37 mm). Note also a cystocele (green line) and a rectal intra-anal intussusception (white arrow). *Red line*: PCL line; *black line*: normal expected contour of the anterior anorectal wall

Rectocele is defined as an abnormal bulge of the rectal wall, most commonly anterior (but also posterior) (Fig. 12), as a consequence of laxity of the rectovaginal fascia [17, 19, 43] and small rectoceles may be entirely asymptomatic in as much as 80% of patients [43]. Physical examination is also unable to accurately measure the dimension of a rectocele [44]. It is considered relevant when surpassing 2 cm beyond the normal expected margin of the anorectal wall during evacuation [17, 19, 35], and it is graded as small, moderate, or severe, when below 2 cm, between 2 and 4 cm and above 4 cm of bulging, respectively [22, 30, 35] (Fig. 13). Rectoceles become important when symptomatic, being responsible for incomplete evacuation and obstruction. Known risk factors include vaginal birth trauma, as well as constipation with chronically increased abdominal pressure [34]. MR defecography, besides being able to grade the rectocele, can also evaluate its emptying dynamic: retention of contrast material after several attempts in the evacuation phase explains the incomplete evacuation.

**Fig. 14:**
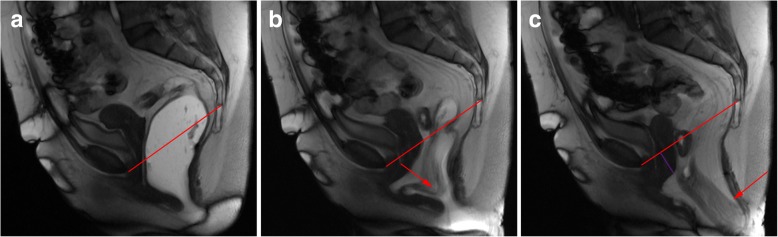
Rectal prolapse with a moderate uterine prolapse. (**a**, **b**, **c**) Sagittal midline TrueFISP images at the beginning (**a**), middle (**b**), and end (**c**) of the evacuation phase demonstrating a rectal intussusception extending beyond the anal sphincter (red arrows). Note also that it is accompanied by a moderate uterine prolapse and a peritoneocele, both aggravating along the different times of the evacuation phase. *Red line*: PCL; *purple line*: extent of the uterine prolapse

Rectal intussusception is characterized by prolapse of full-thickness anorectal wall [45, 46], although mucosa alone may invaginate as well [35, 46]. Rectal intussusception is graded regarding the extent of the invagination as intrarectal, intraanal (Fig. 13b), and extraanal, in the last one being denominated as rectal prolapse, which may be associated with mucosa ulceration and pain [35, 45, 47] (Fig. 14). This entity is responsible for mechanic obstruction with resulting obstipation [45], mostly when the invagination reaches the anal canal. It is relevant when symptomatic or when persistent after defecation. Transient mucosal intussusception during defecation may occur without clinical significance [8]. It is important to differentiate full rectal wall intussusception from mucosa invagination alone, since the surgical approach is quite different [35], and in this point, MR defecography is the only imagiological examination able to accurately discriminate between the two [45]. Also, MR defecography demonstrated that in 30% of cases there is associated descent of the anterior and/or middle compartments in patients with rectal intussusception, which may change the surgical protocol [18] (Fig. 14).

**Fig. 15:**
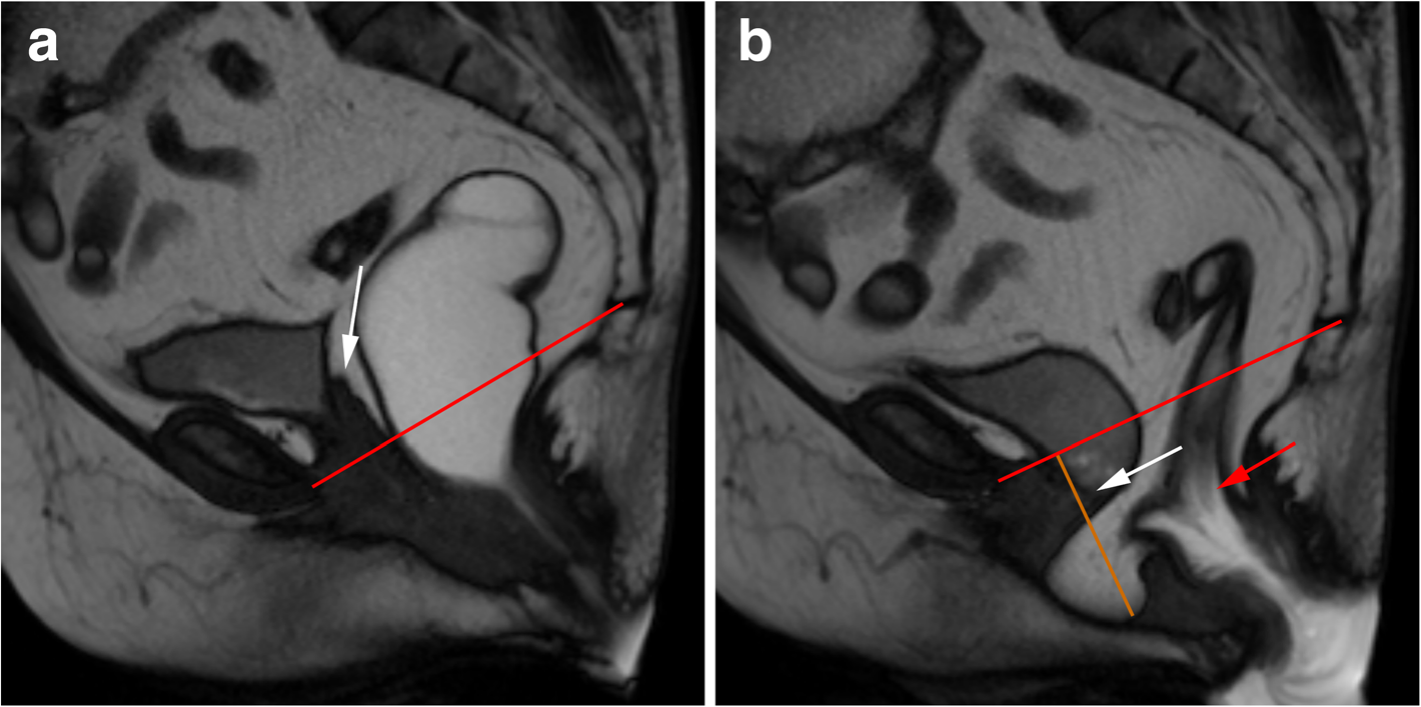
Peritoneocele with vaginal vault prolapse and rectal intussusception. (**a**, **b**) Sagittal midline TrueFISP images at rest (**a**) and during defecation (**b**) of a patient previously submitted to hysterectomy, showing a peritoneocele, where adipose peritoneal folds insinuate to the rectovaginal septum. It is associated with a prolapse of the vaginal vault, with the vaginal apex (white arrows) below the PCL (red line) during the evacuation. An apparent rectal invagination is also present (red arrow). *Orange line*: extent of the peritoneocele

**Fig. 16:**
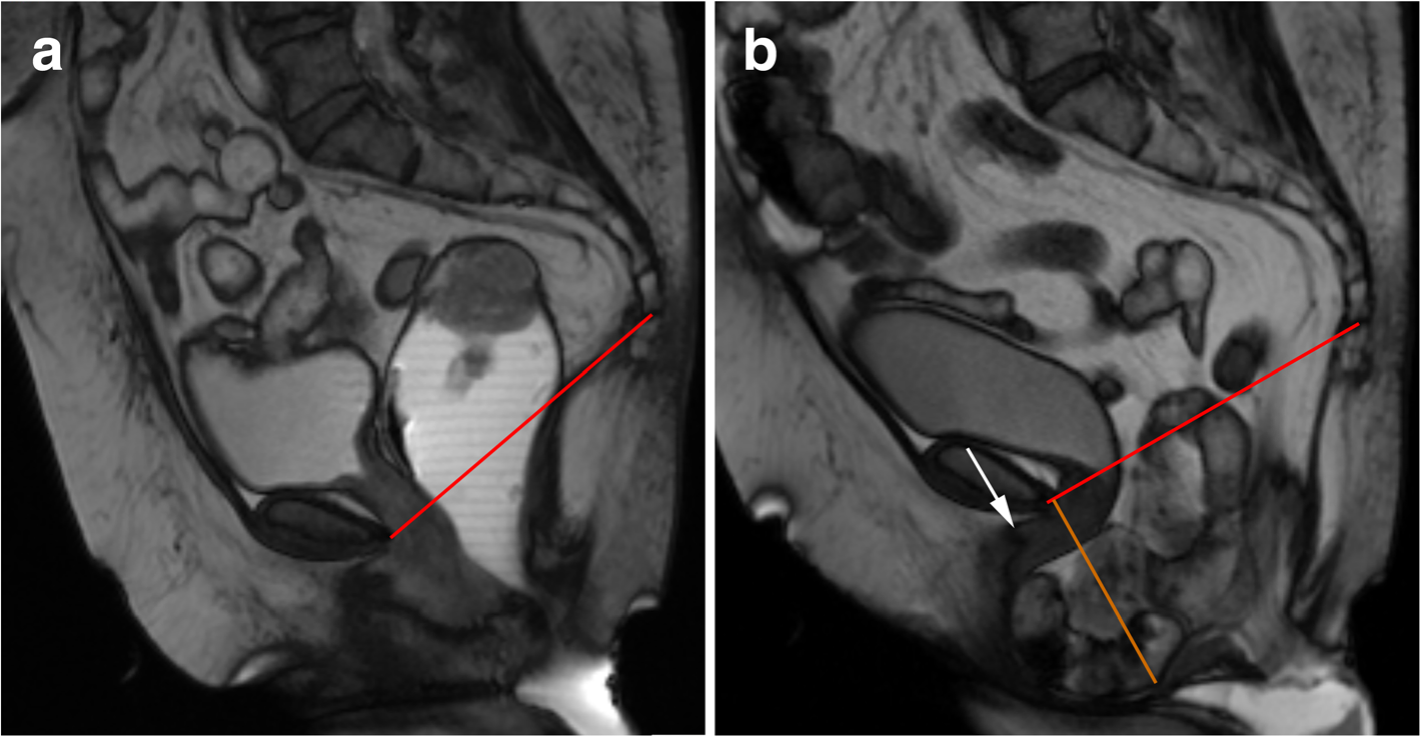
Severe enterocele with vaginal vault prolapse, urethral hypermobility and urinary obstruction. (**a**, **b**) Sagittal midline TrueFISP images at rest (**a**) and during evacuation (**b**) of a patient previously submitted to hysterectomy, showing an enterocele. Note the herniation of small bowel loops into the rectovaginal septum. It is difficult to identify correctly the vaginal apex, but it is clear it is located below the PCL (red line), corresponding to a vaginal vault prolapse. The volume of the enterocele is so large that its mass effect over the vagina and urethra pushes them anteriorly, inducing also an horizontalization of their normal vertical axis—urethral hypermobility (white arrow)—and inducing obstruction of the bladder (the urinary volume of the bladder on both phases is similar, always above the PCL). Note also that the bladder is more distended than it should be. Although not relevant for this specific case, the bladder should be half distended, as preconized by the ESUR/ESGAR recommendations. *Orange line*: extent of the enterocele

Peritoneocele is considered a herniation of peritoneal folds into the rectovaginal septum, below the cul-de-sac, passing the proximal one third of the vagina [35, 46] (Fig. 15). Peritoneoceles are the most difficult pelvic prolapses to be identified during physical examination alone [32]. They are clinically variable, depending mainly on the location and extent of the herniation, but usually present as constipation due to outlet obstruction, incomplete evacuation and a pelvic heavy feeling [34]. When it contains small bowel loops, it is named enterocele (Fig. 16), and when sigmoid colon herniates, it is known as sigmoidocele. As other pelvic prolapses (uterus and bladder), it is graded as small, moderate, or large, when they extend up to 3 cm, 3 to 6 cm, or more than 6 cm below the PCL. MR defecography, unlike cystocolpodefecography, can easily depict the nature of the herniated contents. Patients with a history of hysterectomy are at bigger risk, as well as patients submitted to previous urethropexy [8], and these entities indicate damage to the rectovaginal fascia. On MR defecography, these conditions usually are best depicted at the end of defecation, once the rectum empties and the rectovaginal space enlarges, while intraabdominal pressure intensifies and pushes downward the peritoneum and/or bowel loops. Sometimes, these herniations do not reduce spontaneously, known as perineal hernia.

### Pelvic floor functional disorders

Functional disorders are described separately because, as stated above, they can clinically and radiologically present independent from pelvic organ prolapses [32]. Also, these functional disorders frequently involve all three pelvic compartments at once since, as mentioned, the pelvic floor functions as a single unit, giving support to the entire pelvic structures [9, 11]. Note that, although urethral hypermobility is considered a functional disorder, it was included in the anterior compartment disorder section. The reason is based solely on the described association between a cystocele and urethral hypermobility, which almost never is present alone. Also, some imaging cases of functional disorders in men will be presented, once they are the most common pelvic floor pathology of this gender.

**Fig. 17:**
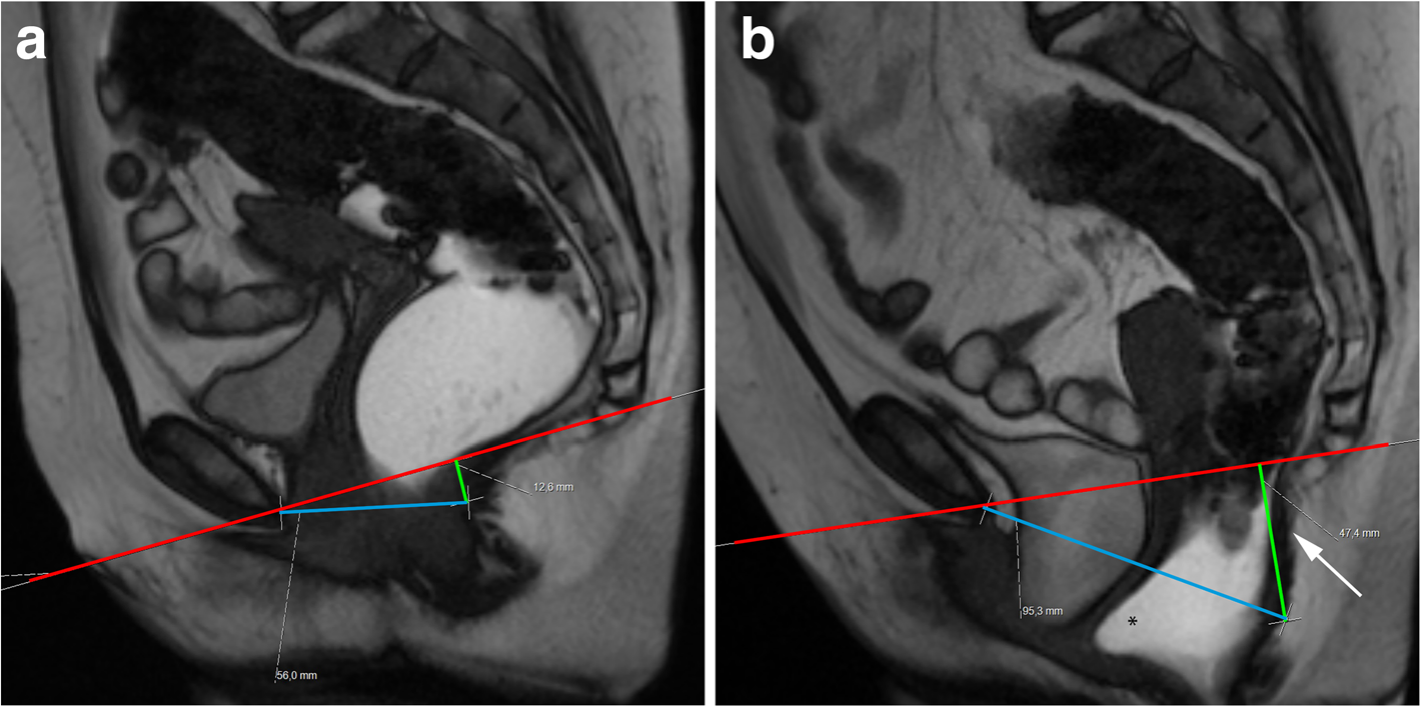
Descending perineal syndrome with tricompartimental prolapse and anterior rectocele. (**a**, **b**) Sagittal midline TrueFISP images at rest (**a**) and during evacuation (**b**). H line (blue line) measures 5.6 cm (**a**) and 9.5 cm (**b**) and the M line (green line) measures 1.3 cm **(a)** and 4.7 cm **(b)**, demonstrating widening and descent of the levator hiatus. Note the vertical orientation of the levator plate (white arrow). It is also associated with a tricompartimental prolapse and an anterior rectocele (*)

**Fig. 18:**
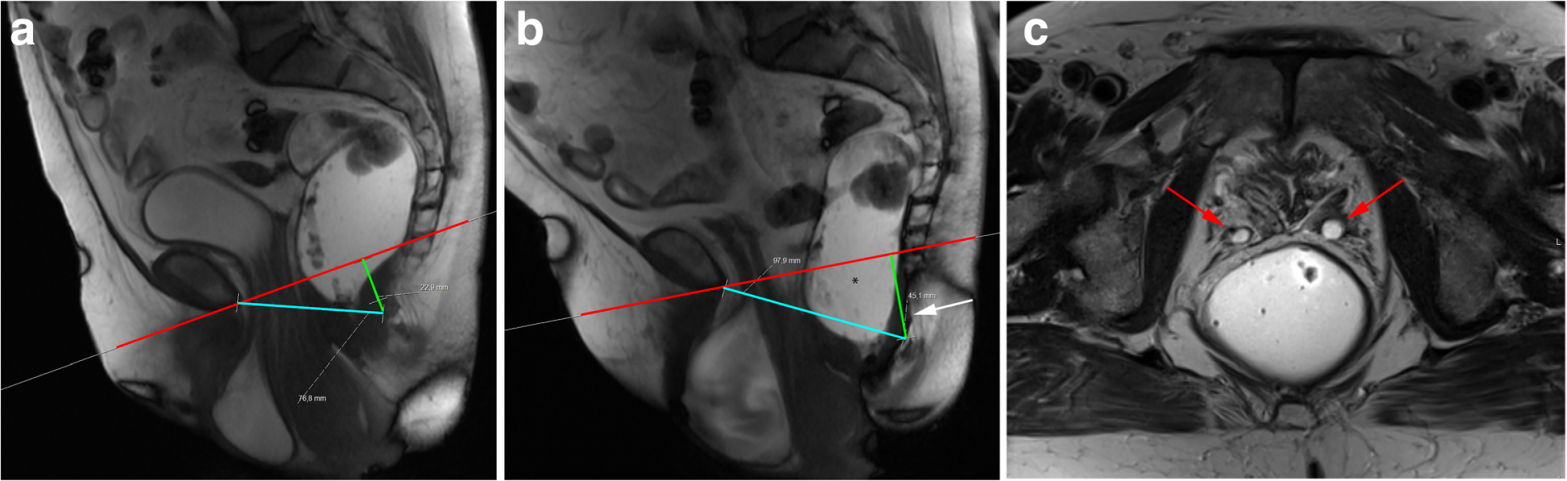
Descending perineal syndrome with tricompartimental prolapse. (**a**, **b**) Sagittal midline TruFISP images at rest (**a**) and during evacuation (**b**). H line (blue line) measures 7.7 cm (**a**) and 9.8 cm (**b**) and the M line (green line) measures 2.3 cm (**a**) and 4.5 cm (**b**), demonstrating widening and descent of the levator hiatus, even at rest but aggravating during defecation. It is also associated with tricompartimental prolapse, with a severe cystocele and a severe uterine everted prolapse. Note the verticalization of the levator plate (white arrow). Another interesting finding is that the rectal contrast is only partially expelled (**b**), because the mass effect by both the cystocele and uterine prolapse induces mechanical posterior obstruction (dilated rectum filled with ultrasound gel) causing constipation and incomplete evacuation (*). (**c**) Axial T2-TSE image at the pubic symphysis plane showing bilateral ureterohydronephosis causes by the cystocele (red arrows)

**Fig. 19:**
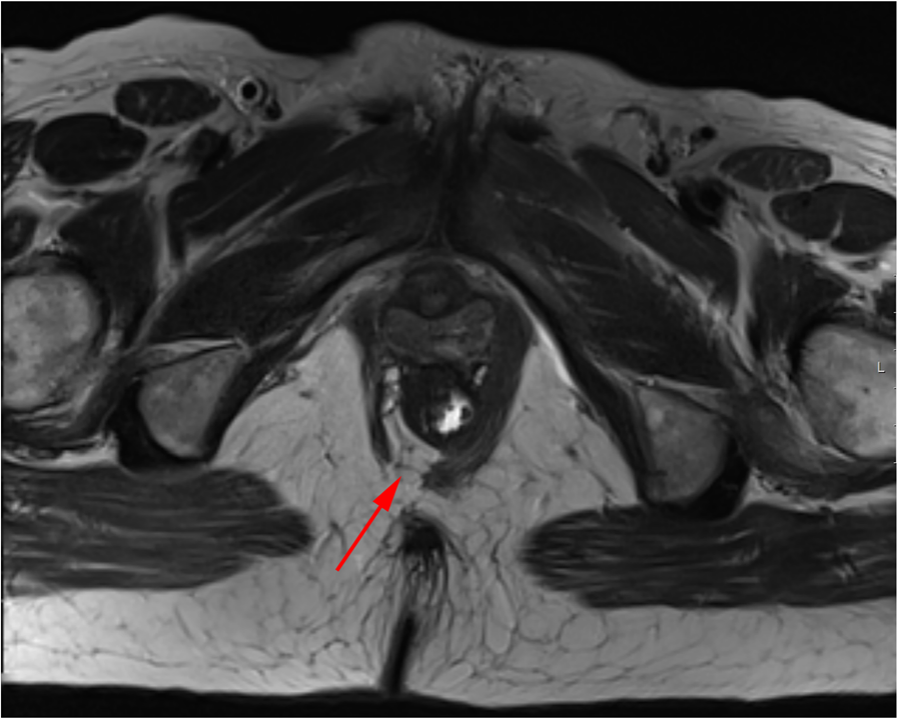
Puborectalis defect. Axial T2-TSE image of the puborectalis muscle, demonstrating a right posterior defect, asymmetry, and atrophy, with lipomatous involution (red arrow)

**Fig. 20:**
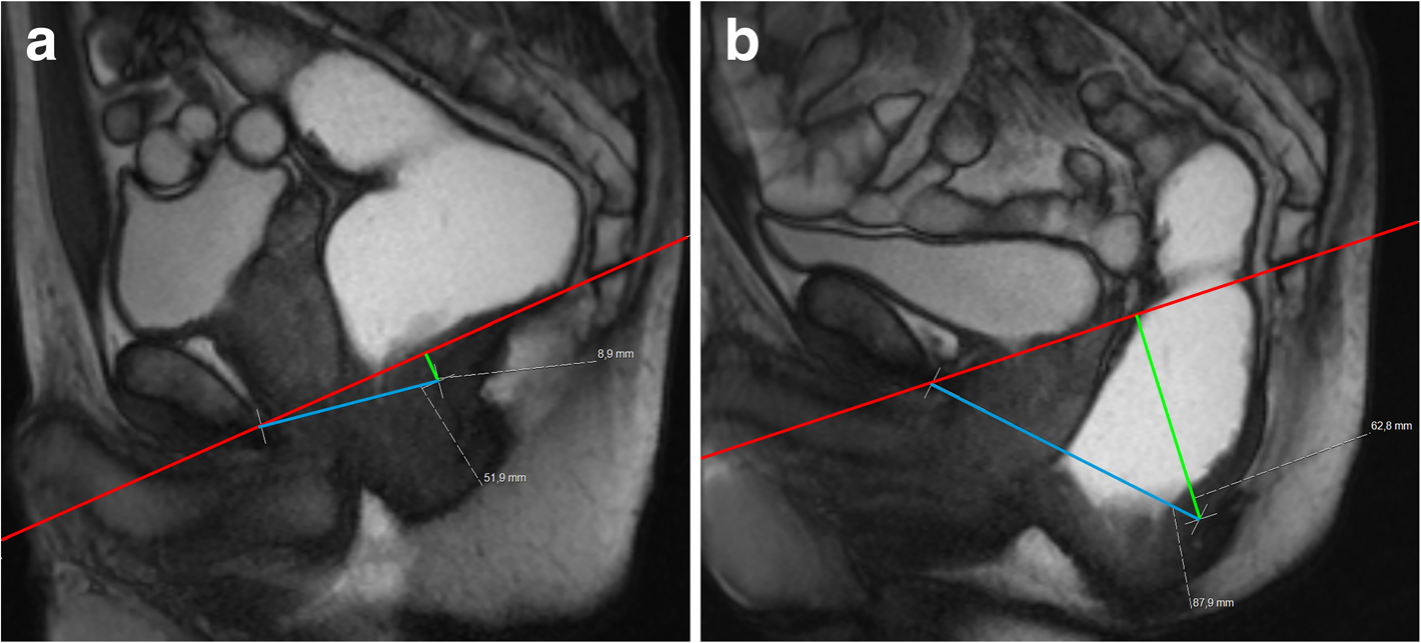
Descending perineal syndrome in a man. (**a**, **b**) Sagittal midline TrueFISP images at rest (**a**) and during evacuation (**b**) of a man with complaints of constipation and incomplete evacuation showing an increase of the H and M lines during evacuation phase (blue line, 88 mm; green line, 63 mm respectively), compatible with descending perineal syndrome with increased hiatal width and descent

Descending perineal syndrome is a condition characterized by loss of tone of the pelvic diaphragm [1, 35, 45] that develops over time. The outcome is excessive descent of the entire pelvic floor at rest and/or during straining and evacuation (Figs. 17 and 18), resulting in the feeling of incomplete evacuation and constipation, which consequently induces increased straining during evacuation and leads to additional neuropathic injury that, in the end, may result in incontinence [34]. Causes for descending perineal syndrome include pudendal nerve injury (from previous delivery trauma or neuropathy), chronic straining at defecation or dysfunctions of the perineal body or levator ani muscles [35]. On MR defecography, suspicious findings include low level of the anorectal junction (increased M line), which is an indicator of muscular tone [1], inability or reduced elevation of the pelvic floor during maximal contraction [45], and associated increased anorectal angle at rest with the inability to normally reduce the angle during squeezing. The levator plate is also usually more vertically oriented [33] (Figs. 17 and 18). Other findings that may be associated include bulging or focal asymmetry of the iliococcygeus or puborectalis muscles [35] (Fig. 19). H and M lines are used to quantify the grade of this condition, since width of the pelvic hiatus is increased. This condition usually involves all pelvic compartments and is frequently associated with pelvic prolapses. In men, it mainly affects the posterior compartment (Fig. 20).

**Fig. 21:**
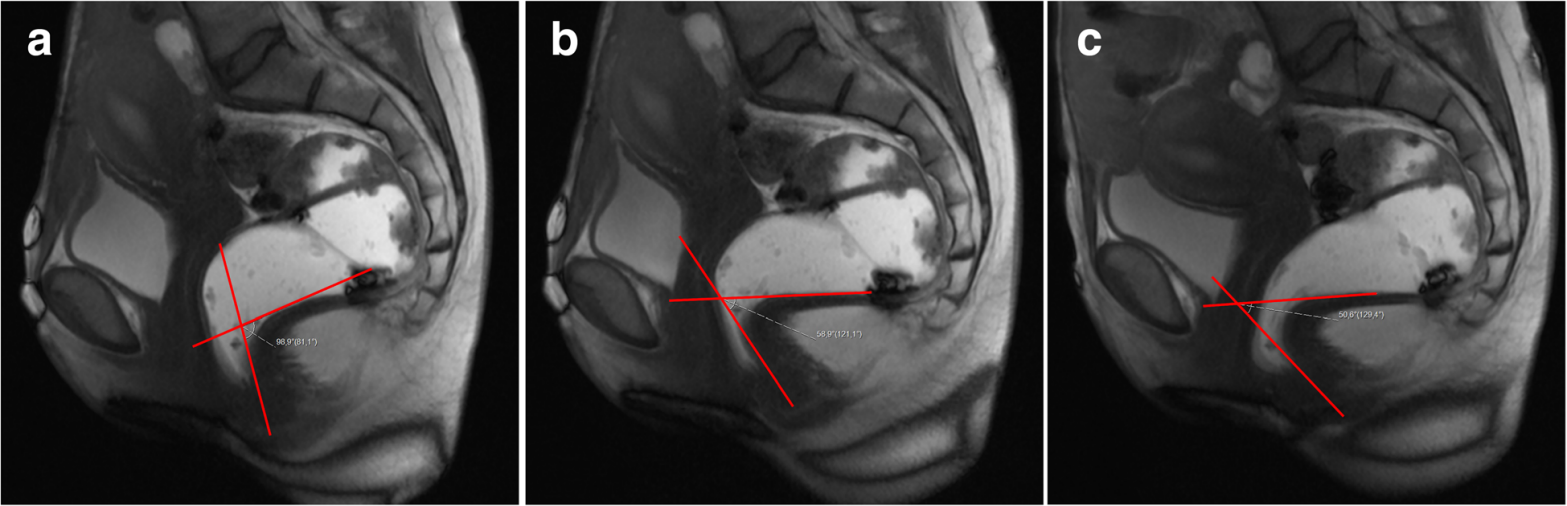
Spastic pelvic floor syndrome. (**a**, **b**, **c**) Sagittal midline TrueFISP images of a 39-year-old patient at rest **(a)**, during squeezing **(b)** and during evacuation **(c)**. Note in **(a)** the ARA is 99° (elevated basal tonus), associated with the reduction of the ARA during defecation (**c**) with 50°, corresponding to a paradoxical contraction of the puborectalis muscle

**Fig. 22:**
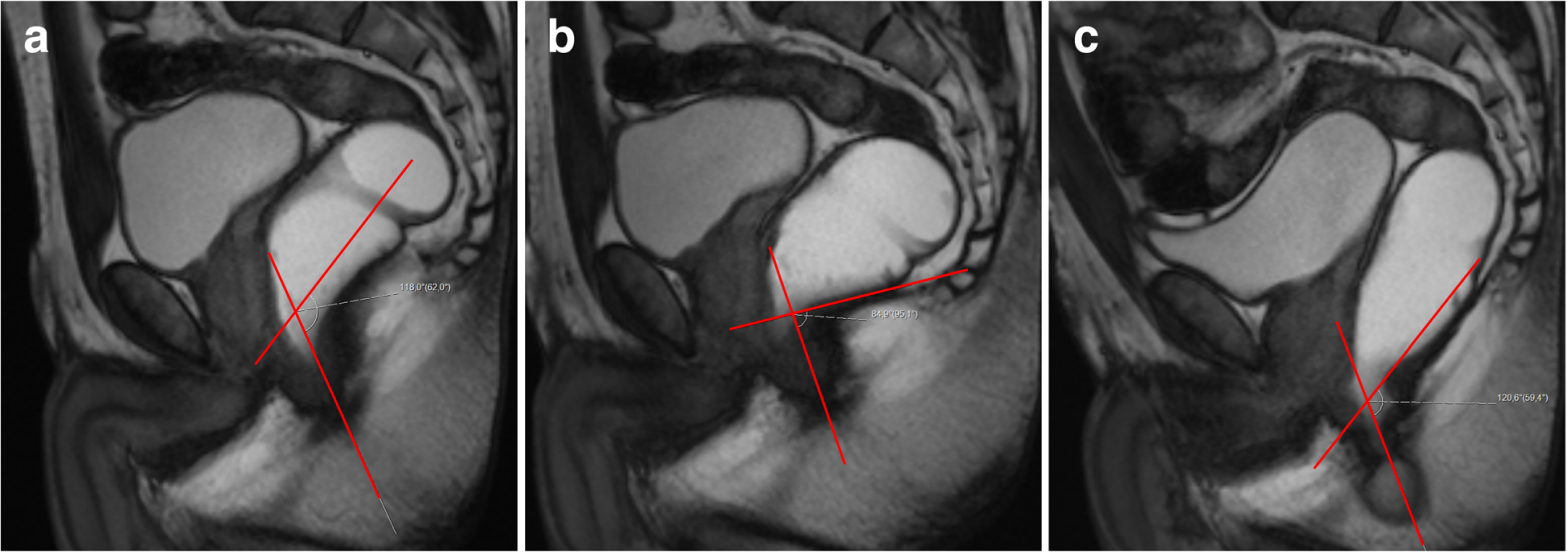
Spastic pelvic floor syndrome. (**a**, **b**, **c**) Sagittal midline TrueFISP images of a man at rest (**a**), during squeezing (**b**) and evacuation (**c**), demonstrating a normal ARA angle at rest in (**a**) (118°), but revealing an increased reduction during squeezing in (**b**) (85°) and a lack of normal widening in the evacuation phase (**c**) (120°). During the dynamic evaluation, spastic contractions of the puborectalis muscle during evacuation were noted as well, and an incomplete evacuation with retention of ultrasound gel was present, all signs compatible with paradoxical contraction of the puborectalis muscle

Spastic pelvic floor syndrome, also known as paradoxical contraction of puborectalis muscle, pelvic floor dyssynergia or anismus, is characterized by involuntary, inappropriate, and paradoxical contraction of the puborectalis muscle during defecation [45, 48], leading to constipation and failed evacuation. Typically, on MR defecography, there is no widening of the anorectal angle (sometimes with shortening) and a descent of the pelvic floor during defecation may or may not be evident [35] (Figs. 21 and 22). This results in a long interval between the opening of the anal canal and the start of defecation, associated with prolonged and incomplete evacuation, the most appropriate findings for the diagnosis of anismus [46]. The puborectalis muscle may appear hypertrophied. Anterior rectoceles are a frequent associated finding [34].

**Fig. 23:**
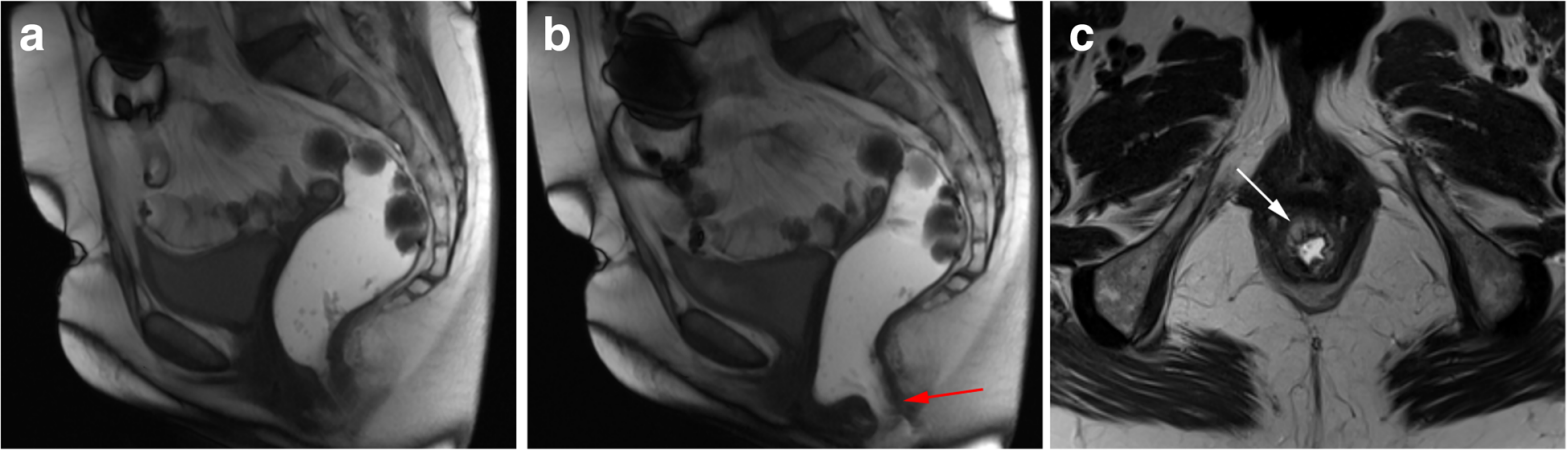
Anal incontinence. (**a**, **b**) Sagittal midline TrueFISP images at rest (**a**) and during Valsava maneuver (**b**), with loss of the ultrasound gel in the second one. Notice the opening of the anal sphincter (red arrow). (**c**) Axial T2-TSE image of the internal anal sphincter demonstrating atrophy with lipomatous involution of the sphincter muscles (white arrow)

Anal incontinence is most of the times caused by direct sphincter laceration or indirect nerve damage from previous vaginal delivery, although neuropathy and iatrogenic damage are also relevant causes [49]. Unaware fecal leakage is usually associated with internal sphincter abnormality, while urge incontinence suggests damage to the external anal sphincter [35]. For anal incontinence alone, MR defecography has little to offer besides showing inability to retain the rectal contrast (Fig. 23). For anatomic sphincter tear or atrophy detection, pelvic MR or ultrasonography are better suited [21], and for functional evaluation, anal canal manometry is the gold standard. However, studies have shown that, in the presence of fecal incontinence, MR defecography is able to change the surgical approach in up to 67% of patients [11], since fecal incontinence may also be the result of end-stage descending perineal syndrome (showing leakage of contrast, anorectal angle changes, pelvic floor descent and rectocele) or intussusception, which justifies the use of MR defecography during the study of these patients.

## Conclusion

Pelvic floor disorders are frequent debilitating conditions, most of the times being targeted for surgical treatment. Since it is frequent to involve more than one pelvic compartment, imaging has a preponderant role in accessing pelvic floor disorders. MR defecography, both with static anatomic and dynamic physiologic sequences, presents as a reliable option for noninvasive pelvic evaluation, being able to ascertain the entire pelvic floor, for optimal patient management before surgery.
